# HTLV-1 gene expression by individual infected clones determines susceptibility to lysis by cytotoxic T lymphocytes specific for Tax and HBZ

**DOI:** 10.1186/1742-4690-11-S1-O21

**Published:** 2014-01-07

**Authors:** Aileen G Rowan, Amy McCallin, Heather A Niederer, Silva Hilburn, Lucy Cook, Graham P Taylor, Charles RM Bangham

**Affiliations:** 1 Section of Immunology, Imperial College London, UK; 2 Section of Infectious Diseases, Imperial College London, UK

## 

Host cytotoxic T-lymphocyte (CTL) responses are critical in limiting expansion of HTLV-1-infected CD4+T-cells in vivo, and most individuals generate abundant Tax-specific CTL. Although Tax is immunodominant, the ability to efficiently present peptides from HBZ on MHC class 1 is associated with a lower proviral load and a reduced frequency of HAM/TSP. HBZ mRNA is expressed in vivo, directing proliferation of infected cells. However, HBZ-specific CTL were detectable in fresh PBMCs in only 25% of chronically infected individuals. We hypothesised that HBZ has evolved to evade the generation of effective HBZ-specific CTLs. To evaluate the selective capacity of a potent HBZ-specific CTL response, we assayed the ability of equally efficient Tax-and HBZ-specific CTL clones to kill unstimulated, naturally infected cells from 16 HLA-A*02+HTLV-1+ individuals. Infected cells which expressed Tax during the course of the assay upregulated surface expression of HLA-A*02, and were eliminated efficiently by Tax-specific CTL. HBZ-specific CTL killed Tax+ cells less efficiently, preferentially killing cells with high levels of HLA-A*02. As a proportion of infected cells do not express Tax owing to silencing, mutation or viral integration site (IS) location, we tested whether HBZ-specific CTLs could kill Tax- infected cells, using high-throughput sequencing to monitor survival of infected clones after CTL selection. We are now validating our findings using patient-derived HBZ-specific CTL, and mapping HBZ epitopes recognised in vivo. In conclusion, the efficacy of HBZ-specific CTLs appears to be limited by the level of antigen presentation, but may confer the ability to target infected cells which escape Tax-specific CTL.

